# Coronary Microcirculation in Aortic Stenosis: Pathophysiology, Invasive Assessment, and Future Directions

**DOI:** 10.1155/2020/4603169

**Published:** 2020-07-22

**Authors:** Jo M. Zelis, Pim A. L. Tonino, Nico H. J. Pijls, Bernard De Bruyne, Richard L. Kirkeeide, K. Lance Gould, Nils P. Johnson

**Affiliations:** ^1^Department of Cardiology, Catharina Hospital, Eindhoven, Netherlands; ^2^Department of Biomedical Engineering, Eindhoven University of Technology, Eindhoven, Netherlands; ^3^Department of Cardiology, Cardiovascular Center Aalst OLV Hospital, Aalst, Belgium; ^4^Department of Cardiology, Lausanne University Hospital, Lausanne, Switzerland; ^5^Weatherhead PET Center, Division of Cardiology, Department of Medicine, McGovern Medical School at UTHealth and Memorial Hermann Hospital, Houston, Texas, USA

## Abstract

With the increasing prevalence of aortic stenosis (AS) due to a growing elderly population, a proper understanding of its physiology is paramount to guide therapy and define severity. A better understanding of the microvasculature in AS could improve clinical care by predicting left ventricular remodeling or anticipate the interplay between epicardial stenosis and myocardial dysfunction. In this review, we combine five decades of literature regarding microvascular, coronary, and aortic valve physiology with emerging insights from newly developed invasive tools for quantifying microcirculatory function. Furthermore, we describe the coupling between microcirculation and epicardial stenosis, which is currently under investigation in several randomized trials enrolling subjects with concomitant AS and coronary disease. To clarify the physiology explained previously, we present two instructive cases with invasive pressure measurements quantifying coexisting valve and coronary stenoses. Finally, we pose open clinical and research questions whose answers would further expand our knowledge of microvascular dysfunction in AS. These trials were registered with NCT03042104, NCT03094143, and NCT02436655.

## 1. Introduction

The seminal 4-group classification of coronary microvascular dysfunction proposed in 2007 placed aortic stenosis (AS) into a category with other myocardial diseases, both primary and secondary [1]. The importance of microcirculatory dysfunction due to AS has become even more clear given the confluence of increasing prevalence due to demographic changes [2] and of expanding treatment since the development of transcatheter aortic valve implantation (TAVI) [3]. Nevertheless, clinical observations enabled by refined diagnostic testing and less invasive treatment have, if anything, exposed unresolved physiologic questions regarding how we should understand, assess, and manage microvascular dysfunction in the patient with AS. This review addresses this practical need by summarizing the hemodynamic pathophysiology linking aortic stenosis and myocardial dysfunction, describing our invasive tools for quantifying microcirculatory function including its relationship with epicardial stenosis, and noting unresolved questions of clinical importance and how they might be answered. For clarity, we only focus on AS without coexisting myocardial pathology like amyloid or other infiltrative diseases.

## 2. Supply versus Demand

Uniquely among our organs, the heart must pump its own blood supply and cannot meaningfully augment oxygen extraction, implying that only increased supply can match increased need. Wall stress, contractility, and heart rate account for most myocardial oxygen consumption. The law of Laplace informs us that wall stress is directly proportional to pressure and to radius but inversely proportional to thickness. AS increases wall stress through elevated afterload, and, in response, the heart compensates through increased wall thickness. In other words, left ventricular (LV) hypertrophy offsets pressure overload to reduce wall stress and thereby oxygen requirements. However, LV hypertrophy brings its own disadvantages, namely, diastolic dysfunction, insufficient capillary density [4], and diffuse fibrosis [5].

As a semiquantitative and practical metric of coronary supply versus myocardial demand, a unitless index has been proposed using pressure measurements [6]. The area under the aortic (or, in situations of aortic stenosis, LV) curve during systole (the so-called systolic pressure time integral or SPTI) has been shown in animal models to have a very high and direct correlation with myocardial oxygen demand, even superior to the pressure-rate product [7]. The area between the aortic (or, in situations of epicardial disease, distal coronary) and LV pressure curves during diastole (the so-called diastolic pressure time integral or DPTI) provides a more sophisticated but similarly motivated metric than “coronary perfusion pressure” (difference in end-diastolic pressures between the aorta and LV) and resembles the supply to the myocardium. DPTI/SPTI balances supply and demand into a single unitless ratio, although this formulation ignores other factors such as arterial oxygen content and relative LV mass and wall tension [6]. Directional changes in an individual patient signal dynamic shifts in supply versus demand, while its unitless adjustment for absolute heart rate and blood pressure variation enables cross-sectional comparison among patients.

Although commonly considered as a single “myocardium,” the subepicardial and subendocardial layers display distinct patterns of blood flow with differential sensitivity to pathology. On the one hand, the subepicardium faces generally low pressures from the pericardial space and thoracic cavity throughout the cardiac cycle, while, on the other hand, the subendocardium experiences generally low LV filling pressures during diastole that rise dramatically during systole. Even under normal conditions, the LV pressures during systole compress the subendocardium and redistribute flow to midmyocardial and subepicardial layers [8], a phenomenon explained by competing ‘vascular waterfall' [9] and ‘intramyocardial pump' [10] models. Consequently, after a 90-second coronary occlusion, the subepicardium reperfuses more quickly than the subendocardium [11]. Furthermore, during a wide range of pathologic perturbations, “the decrease in subendocardial and increase in subepicardial flow were often associated with normal or even elevated total coronary blood flows” [12], indicating that transmural maldistribution provides a unique guide for understanding many disease states. In animal models, a ratio below 0.8 has been demonstrated via microspheres to correlate with a reduction in subendocardial flow relative to the subepicardium; values >0.8 have been associated with intact and relatively homogeneous perfusion among myocardial layers [6].

**Figure 1:**
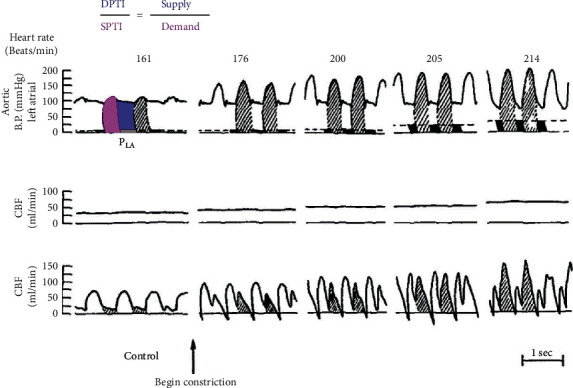
Animal aortic banding model that parallels the development of aortic valvular stenosis: at baseline, the systolic demand (shaded) and diastolic supply (not shaded) are well balanced when recording the aortic and left atrial pressures in this animal model of dynamic, supravalvular stenosis. With progressive banding demand rises (shaded area increases), supply falls (due to acute tachycardia in this animal model but also rising left atrial filling pressures marked as filled areas during diastole). Coronary blood flow (CBF, which corresponds to mean coronary blood flow) begins as diastolic dominant (unique to the normal heart) but concludes as systolic dominant (more typical of a peripheral organ bed) (reprinted from Figure 2 of a 1972 publication [12]).

To apply these principles of supply versus demand to aortic stenosis, consider the animal model in Figure 1 [12]. Under control conditions, no gradient exists between the LV and the aorta, DPTI and SPTI have similar areas under their respective curves (for a supply/demand ratio close to unity), and coronary perfusion displays a diastolic dominant pattern. As constriction begins using a band around the ascending aorta, left atrial pressure (a surrogate for LV filling pressures) rises, reducing DPTI supply at the same time that an elevated systolic pressure increases SPTI demand. Coronary flow becomes more dependent on flow during systole. With progressive constriction, these changes continue with falling DPTI supply (through a combination of increasing left atrial pressure and tachycardia), rising SPTI demand (as the band creates an ever worse supravalvular aortic stenosis), and emerging systolic-dominant coronary flow pattern. This fall in DPTI/SPTI preferentially affects the subendocardium; other animal studies have demonstrated a uniform endocardial/epicardial ratio of 0.97 and flows above 6 cc/min/g under normal hyperemic conditions but an imbalanced ratio of 0.80 (less subendocardial flow) and fall in flow to below 4 cc/min/g with valvular AS [13].

In many ways, Figure 1 provides a conceptual template for what happens in humans, albeit over a different time scale. Progressive AS increases SPTI, while rising LV filling pressures decrease DPTI, leading to a net reduction in the supply/demand (DPTI/SPTI) ratio. However, acute banding in animals does not have time to produce LV hypertrophy as in humans, which further increases the vulnerability of our subendocardium. Also, note that the tachycardia from acute banding in an animal model does not occur in humans with slowly progressive disease, although it represents an additional mechanism for reducing DPTI. For example, in a human cohort, with normal angiograms but critical aortic stenosis (4 subjects, mean gradient 93 mmHg, and aortic valve area 0.48 cm^2^), undergoing invasive hemodynamic study, average DPTI/SPTI of 0.34 with net lactate extraction at baseline 85 beats/minute fell with isoproterenol stress to DPTI/SPTI of 0.16 and switched to net lactate production at 113 beats/minute [14]. These observations could explain why patients can have angina from AS even with normal coronary arteries [15].

## 3. Myocardial Resistance

Unfortunately, our intuitive notion of “resistance” gained from daily life and basic electrical circuits often provides a suboptimal analogy for understanding myocardial behavior. As a result, much of the literature on “myocardial resistance” must be reviewed with caution or at least through the lens of a more sophisticated understanding. This section discusses key points relevant to understanding the concept as it applies to AS since the general topic goes beyond the scope of this review.

During baseline or resting conditions, myocardial flow remains relatively stable over a wide range of perfusion pressures [16] via a large number of homeostatic control mechanisms referred to in aggregate as “autoregulation.” Consequently, basal myocardial resistance represents a dynamic phenomenon without unique value. Only under conditions of vasodilation does a largely linear relationship exist between perfusion pressure and flow, although somewhat curvilinear at very low perfusion pressures below the range of stable patients. The slope of this hyperemic relationship can be used to estimate resistance. However, in crucial distinction to an electrical resistor, coronary pressure does not fall to 0 mmHg with complete occlusion of the epicardial artery. Depending on how it is measured, this residual pressure has been termed the coronary “wedge pressure” or “zero-flow pressure” or “back pressure.” When accounting for venous and aortic pressures, the scaled wedge pressure quantifies relative maximum collateral blood flow [17].

**Figure 2:**
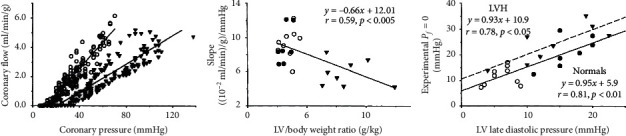
Myocardial resistance in an animal model of aortic stenosis: at about 2 months of age, a 20–25 mmHg peak systolic gradient is created in dogs who were then studied at 10–14 months of age and compared with normal animals. During intravenous adenosine infusion, coronary flow is measured as a function of coronary pressure with progressive coronary constriction. Open circles represent normal dogs, and closed triangles represent those with supravalvular aortic stenosis. The flow versus pressure relationship (left) shifts to the right and rotates clockwise when moving from normal to aortic stenosis. Its slope relates inversely to the amount of left ventricular hypertrophy (middle), indicating a dose-response relationship. Its intercept correlates directly with left ventricular filling pressures (right). In these ways, the decrease in slope corresponds to an increase in myocardial resistance and the change in intercept to a rising zero-flow pressure due to higher LV filling pressures (reprinted from Figures 1–3 of a 1993 publication [19]).

Animal models of supravalvular aortic stenosis inform us about its effects on myocardial resistance. Compared to normal dogs, animals with LV hypertrophy after 8–10 months of aortic banding displayed a more shallow slope (less flow for the same coronary pressure) but also a higher wedge pressure [18] as depicted in Figure 2. More LV hypertrophy was associated with shallower slopes in that study, implying a dose-response relationship. Additionally, the wedge pressure was roughly twice as high in the setting of LV hypertrophy (24 mmHg versus 12 mmHg) and correlated with LV filling pressures (Pearson coefficient approximately 0.8, indicating that 0.8^2^ = 64% of the variation can be explained).

Several aspects add further complexity to this vasodilated relationship between flow and pressure. First, inotropic (dobutamine and exercise) and chronotropic stimulation can change the slope by about 20% in addition to increasing the wedge pressure [19, 20]. This change in slope, corresponding to a higher resistance, might reflect the compressive effects of higher LV pressure and/or relatively more time spent in systole, indicating that a unique “minimum resistance” cannot be expected. Second, the myocardium displays capacitive and inductive effects necessitating the more general concept of impedance to account for phasic aspects in aortic pressure and flow. While many publications describe diastolic pressure/flow relationships [21], few account for these active effects that largely average out over the entire cardiac cycle. Third, the subepicardium and subendocardium display different pressure/flow relationships, generally with a similar slope but a lower zero-flow pressure in the subepicardium [22].

Before presenting existing resistance data in humans with AS, several points deserve to be mentioned. First, two main invasive techniques exist to measure coronary flow (Doppler flow velocity and bolus thermodilution), thereby introducing heterogeneity in the literature. Encouragingly, vasodilatory hyperemia to enable either technique appears safe in patients with severe AS based on 40 reports from 1820 patients over 3 decades as summarized in Table 1. Second, techniques using bolus thermodilution [62] and Doppler flow velocity [63] have demonstrated an important bias when quantifying resistance by neglecting wedge pressure assessment in situations when the wedge pressure is elevated. Since most patients with severe AS undergoing TAVI or surgical aortic valve replacement (SAVR) will have at least a moderate elevation in LV filling pressure, which tracks with wedge pressure [18, 19], resistance measurements without this correction should be viewed skeptically. Third, to our knowledge, no study has yet distinguished between changes in wedge pressure versus slope when studying myocardial pressure/flow relationships in human AS. However, continuous thermodilution with the added technique of proximal balloon inflation can create an almost continuous flow versus pressure curve that allows both parameters to be estimated [64].

Table 2 presents a summary of the literature that has reported resistance assessment in humans with AS, both before and after TAVI. Data from 7 studies with a total of 174 vessels either compared resistance between normal patients and those with severe AS and/or serial resistance measurements in the same patients with severe AS before and after TAVI. While limited by modest sample sizes, two different techniques for measuring resistance, and lack of separate slope and zero-flow pressures (apart from 1 study that did measure wedge pressure explicitly), the data suggest two key points in keeping with the animal work described previously: myocardial resistance in AS exceeds that in normal subjects, and resistance falls after TAVI, both acutely and in the longer-term.

## 4. Epicardial Stenosis

While severe AS by itself can be sufficient to explain symptoms of heart failure or angina, due to a supply-demand mismatch discussed above, epicardial coronary disease of angiographic significance can be seen in 40% to 75% of these patients [65]. Due to near-ubiquitous coronary angiography before TAVI, either invasively or via computed tomography, frequently identified epicardial lesions pose an unresolved treatment dilemma. Rarely is a stenosis so proximal and critical as to require percutaneous coronary intervention (PCI) in order to perform TAVI safely. In most situations, a stenosis could be treated either before or after TAVI with tradeoffs among benefit (usually symptoms resolve with TAVI alone, and the impact of PCI on spontaneous myocardial infarction remains unclear in this older population with severe AS), ease of coronary access (more difficult after TAVI), periprocedural risk (potentially, complications are less well-tolerated with severe AS), and antiplatelet therapy (less flexible after PCI).  Table 3 summarizes ongoing randomized trials in this area. In the interim, observational data using fractional flow reserve (FFR) suggested improved outcomes, defined as a composite of death, myocardial infarction, and stroke, versus angiographic selection, mainly through the avoidance of procedural complications in lesions lacking a large hyperemic pressure gradient [66].

**Figure 3:**
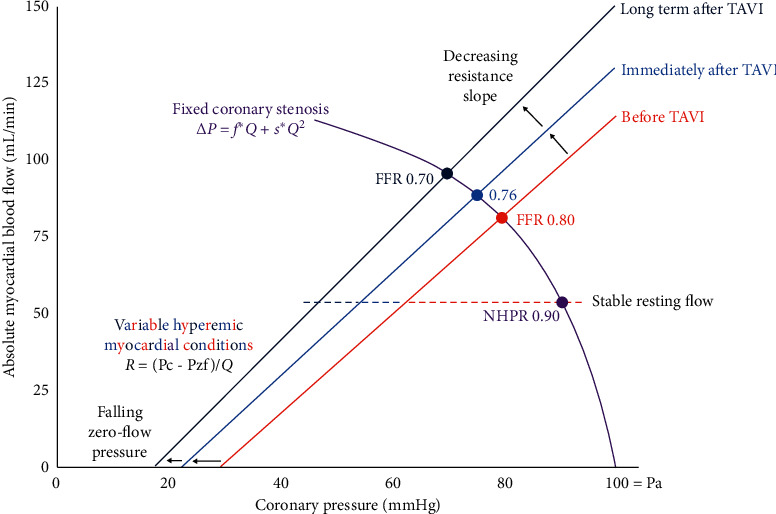
Myocardial flow versus coronary pressure relationships: during hyperemia, a linear relationship exists between absolute myocardial blood flow and coronary pressure (basically equal to aortic pressure in the absence of a stenosis). This so-called myocardial “load line” has both slope (how much extra flow for an increase in driving pressure) and offset (often referred to as the zero-flow or wedge pressure depending on how it is measured). The slope of the myocardial load line corresponds to the myocardial resistance which can be calculated through the formula *R* = (Pc − Pzf)/*Q*, where *R* is the resistance, Pc is the coronary pressure, Pzf is the zero-flow, and *Q* is the flow. Under resting conditions (horizontal dashed line), the myocardium is capable of autoregulation to maintain a roughly constant flow over a wide range of perfusion pressures reflected by a constant nonhyperemic pressure ratio (NHPR). A fixed coronary stenosis produces both friction (“*f* ”) and separation (“*s*”) components to net pressure loss as can be deduced from the well-known coronary stenosis formula ΔP = *f* ∗ *Q* + *s* ∗ *Q*^2^, where *P* is the pressure loss in mmHg and *Q* is the coronary flow in mL/min [67]. Its intersection with the myocardial load line represents the observations of FFR and maximum flow at peak hyperemia. Potential changes in the myocardial load line have been shown before versus after transcatheter aortic valve implantation (TAVI), although the relative magnitude and time course of a left shift (due to a fall in left ventricular filling pressures) and counterclockwise rotation (corresponding to more flow for the same driving pressure) have not yet been quantified (reprinted from the figure of recent 2020 editorial [68]).

Superimposing a coronary stenosis on severe AS exacerbates the supply/demand mismatch. A fixed epicardial stenosis produces a pressure loss that increases with flow but has separate contributions from viscous (friction, linear) and separation (expansion, quadratic) components.  Figure 3 superimposes this net stenosis pressure/flow relationship on the description of myocardial performance during vasodilation. The intersection of the stenosis curve and the myocardial load line represents the observations at hyperemia with corresponding FFR and coronary flow reserve (CFR) values [69]. During resting conditions, coronary flow is controlled by autoregulation and does not change, translating into stable nonhyperemic pressure ratios over time as demonstrated in the literature summarized in Table 4. While constancy can be comforting, it overlooks that most patients remain asymptomatic at rest, and thus, only a hyperemic assessment could link with exertional symptoms, acknowledging that dedicated studies in AS are currently lacking and would be confounded by valvular symptoms.

Based on the discussion of myocardial resistance in the prior section, the existing data support an increase in hyperemic flow after TAVI due to a change in the myocardial load line. This change occurs both via a reduction in wedge pressure, largely mediated by its direct correlation with LV filling pressures [18, 19] that fall after AS has been treated, and a counterclockwise rotation from increasing slope [18]. However, the existence, time course, and relative magnitude of these changes after TAVI in humans have not been demonstrated.

In contrast to inferences regarding the mechanisms in Figure 3, the secondary effect on the intersection of a fixed stenosis curve but dynamic myocardial load line can be seen more directly from observations summarized in Table 4 from 12 publications and about 350 lesions [68]. Overall resting flow may decrease slightly in the first year as expected from reduced myocardial demand, although the data imply that this effect remains modest and has essentially no impact on nonhyperemic pressure ratios. More clearly suggested by the data is an acute increase in peak hyperemic flow with concomitantly higher CFR and lower FFR. However, these studies were small or modestly sized, used a variety of measurement techniques for flow, and did not stratify changes based on properties of the myocardial load line or stenosis curve. In these studies, coronary hyperemia was appropriately induced by pharmacologic stress in order to focus on fixed epicardial disease emphasized by pure vasodilation (and appropriate for revascularization) as opposed to exercise stress that includes vasoconstriction whose treatment is fundamentally medical.

**Figure 4:**
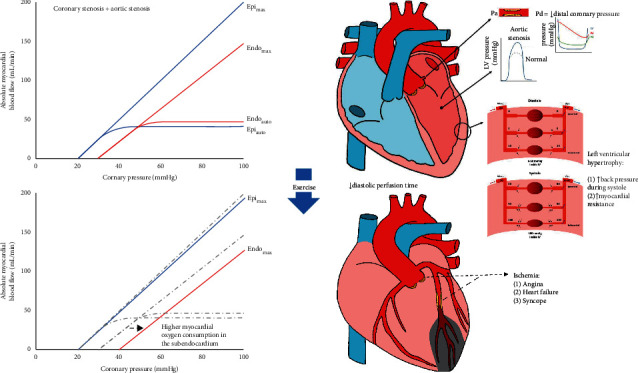
Transmural impact of aortic stenosis with coronary disease: reduced flow from aortic stenosis and coronary stenosis does not affect all layers of the myocardium equally. Under baseline conditions, autoregulation (“auto” subscript) maintains a relatively stable flow for most perfusion pressures. Vasodilation (“max” subscript) produces the net hyperemic myocardial load line from Figure 3 that is made up of a lower offset in the subepicardium (Epi) than the subendocardium (Endo), with potentially different slopes as well. Exercise reduces diastolic perfusion time and increases left ventricular pressures, preferentially affecting the subendocardium both through tachycardia and also increased oxygen consumption. The resulting hypoperfusion can produce the classic symptoms of valvular stenosis.

The proposed model in Figure 3 neglects the important physiologic differences between the subepicardium and the subendocardium. Thus, Figure 4 depicts two separate curves relating pressure and flow in distinct layers of the myocardial wall. Under conditions of vasodilation, the higher LV pressures reduce flow in the subendocardium, which becomes further exacerbated as diastolic perfusion time decreases with exercise. While not possible to measure different FFR values in various layers of the myocardium, Figure 4 nevertheless provides an explanatory framework for understanding the differential impact of epicardial coronary lesions on the microvasculature.

**Figure 5:**
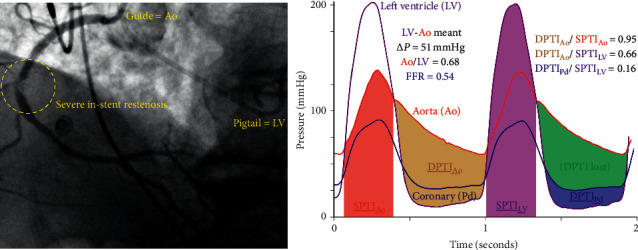
Clinical case of simultaneous aortic and coronary stenosis assessment: as detailed in the text, this 82-year-old man with exertional dyspnea underwent coronary evaluation before transcatheter aortic valve implantation. Three pressures were measured simultaneously: aortic (via the guide catheter), coronary (via a distal pressure wire), and left ventricular (via a pigtail catheter). Intravenous papaverine induced coronary hyperemia with a fractional flow reserve (FFR) of 0.54. Both the severe aortic stenosis (baseline mean gradient 51 mmHg) and the severe in-stent coronary lesion imbalance myocardial demand (systolic pressure time integral, or SPTI) and diastolic coronary supply (diastolic pressure time integral, or DPTI). This figure allows for a visual understanding of the additive effects of the tandem aortic valve and coronary stenosis.

Figure 5 provides a clinical example of applying the DPTI/SPTI concept to individual data from an 82-year-old man with exertional dyspnea and a severe in-stent lesion in the right coronary artery but also a mean aortic valve gradient of 51 mmHg. In this case, an already reduced DPTI/SPTI became radically diminished as a result of diastolic pressure loss from the epicardial lesion. During hyperemia, the FFR reached 0.54, and the DPTI/SPTI fell to 0.16, the same as the average value in the previously mentioned study in which patients with critical AS switched to lactate production [14]. While removing the coronary stenosis might have increased the DPTI/SPTI to 0.66, only treating both AS and the coronary lesion would produce a balanced DPTI/SPTI of 0.95. As noted earlier, we do not have randomized trials demonstrating clinical advantages to treating coexisting coronary disease, but hemodynamically severe and focal lesions supplying large amounts of myocardium, as in this case, seem reasonable candidates for PCI based on using FFR in patients without AS.

## 5. Unanswered Questions

A review not only provides an opportunity to look backward and synthesize existing knowledge but also offers the possibility to identify gaps that remain and how they might be filled in the future. On a basic level, measuring in humans the changes seen in animal models [18] regarding myocardial load lines versus zero-flow pressure would provide us with a better appreciation for acute versus chronic benefits of TAVI. Perhaps, the immediate procedural impact of TAVI on the myocardium predominately affects zero-flow pressure (through a reduction in LV filling pressures as seen in animal work [18]), whereas chronic remodeling over months mainly changes the slope of the myocardial load line (through a regression in LV hypertrophy as seen in animal models [20]). Continuous thermodilution with the added technique of proximal balloon inflation provides perhaps the most comprehensive yet practical examination in order to separate and quantify these effects in actual patients undergoing treatment [64].

Apart from a confirmation of translational animal physiology and conceptual insight, what clinical advantages might come from such data? Currently, we do not understand when to treat mixed coronary disease and AS, and in some cases, FFR values fall after TAVI, particularly when previously in the 0.75 to 0.85 range [68]. If we understood the degree and time course of myocardial changes after TAVI, then we could better predict which coronary lesions might take on added importance and benefit from revascularization versus those that would remain hemodynamically modest, even after longer-term remodeling. Additionally, some patients with valvular cardiomyopathy recover LV function after TAVI, whereas others remain depressed. Does the slope of the myocardial load line predict this potential reversibility? If yes, then it would permit better patient selection in order to optimize the TAVI risk/benefit. Finally, the changes in myocardial resistance should be expected to be linked to pretreatment severity of the AS as well as the hemodynamic efficacy of the TAVI device. Because myocardial resistance is inherently a hyperemic concept, do baseline parameters such as the resting valve gradient or aortic valve area perform worse than hyperemic parameters such as the stress aortic valve index (SAVI) [72]? In that case, it would argue against relying solely on resting measurements when selecting patients for TAVI.

**Figure 6:**
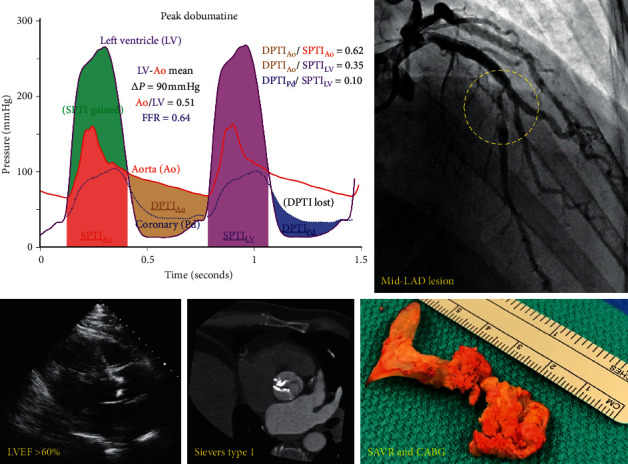
Clinical case of asymptomatic but severe stenosis: as detailed in the text, this 55-year-old asymptomatic man was referred for an incidental heart murmur on routine physical examination. A treadmill exercise test showed good functional capacity with no symptoms or abnormal responses, and echocardiography found normal ejection fraction. However, his bicuspid aortic valve had moderate-to-severe stenosis at baseline, rising to a mean gradient of 90 mmHg during intravenous dobutamine stress. Furthermore, his left anterior descending (LAD) coronary artery had an angiographically moderate-to-severe stenosis and fractional flow reserve (FFR) of 0.64 during intravenous adenosine infusion. When superimposing these curves (the distal coronary pressure tracing has been time-scaled to match the aortic pressure tracing), myocardial oxygen demand (systolic pressure time integral, or SPTI) greatly exceeds diastolic coronary supply (diastolic pressure time integral, or DPTI) due to increased SPTI from aortic stenosis and decreased DPTI due to coronary stenosis. Despite normal left ventricular function and a lack of symptoms, the patient underwent surgical aortic valve replacement (SAVR) and concomitant coronary artery bypass grafting (CABG) for extremely abnormal hemodynamics.

As a final sign of our yet incomplete knowledge of the coronary microcirculation in aortic stenosis, a clinical case is considered in Figure 6. This 55-year-old man was referred by his internist for an incidental murmur noted during a routine physical examination that was otherwise unremarkable. In daily life, he had no symptoms and performed 9:32 minutes of a standard Bruce treadmill protocol. Blood pressure, heart rate, and heart rhythm response were normal during the graded exercise; he denied angina and stopped due to leg fatigue. Echocardiography revealed normal LV function with an ejection fraction over 60%. Therefore, we have an asymptomatic patient with no evidence of subclinical cardiomyopathy.

However, extensive workup revealed a calcified bicuspid aortic valve with moderate-to-severe stenosis at baseline, rising to a mean gradient of 90 mmHg during intravenous dobutamine infusion with a SAVI of 0.51 (indicating that peak valvular flow is reduced by the stenotic valve to 51% of maximum). A calcified mid-LAD stenosis had an FFR of 0.64 during intravenous adenosine with a focal pressure jump. An analysis of DPTI/SPTI (although not direct in this case since the valve and coronary stenoses were interrogated sequentially using different pharmacologic agents) showed a potential drop to 0.10 during peak stress, entering the region that has been associated with net lactate production in a small human study [14]. Therefore, we have coexisting and severe aortic and coronary stenoses confirmed by objective hemodynamic data.

Should we understand this case as a profound challenge to the relevance of hemodynamic physiology reviewed in this article? Or does it indicate that patient symptoms (or their lack) as well as standard noninvasive testing often tell us at most a modest amount regarding physiologic severity, thereby necessitating routine quantification? While awaiting the results of ongoing randomized controlled trials of TAVI in severe yet asymptomatic AS (clinicaltrials.gov, NCT03042104, NCT03094143, and NCT02436655), what should we currently do with such patients who nevertheless exhibit extreme hemodynamic derangements? Can we expect that the reduction in sudden death seen in a small trial of SAVR for asymptomatic yet severe AS (mean gradient 63 mmHg) [73] will be confirmed in larger, ongoing trials? While these vital questions cannot be answered definitively at this moment, they serve as humble reminders regarding the profound capacity of the human coronary microcirculation in some patients to withstand a severe assault on multiple fronts.

## Figures and Tables

**Table 1:** Literature review of vasodilator stress agents in severe aortic stenosis.

Authors	Citation	*N*	Drug	Technique	Safety issues
Roy et al. [23]	*Nucl Med Commun* 1998; 19: 789	12	Dipy	SPECT	No
Carpeggiani et al. [24]	*J CV Med* 2008; 9: 893	15	Dipy	PET	No
Liu et al. [25]	*Sci Rep* 2019; 9: 12443	15	Dipy	SPECT	No
Burwash et al. [26]	*Heart* 2008; 94: 1627	20	Dipy	PET	No but 16 excluded
Rajappan et al. [27]	*Circulation* 2002; 105: 470	20	Dipy	PET	No
Nemes et al. [28]	*Herz* 2002; 27: 780	21	Dipy	TTE	No
Baroni et al. [29]	*Heart* 1996; 75: 492	25	Dipy	TTE	No
Huikuri et al. [30]	*AJC* 1987; 59: 336	27	Dipy	SPECT	2 hypotension
Demirkol et al. [31]	*Cardiology* 2002; 97: 37	30	Dipy	SPECT	No
Nemes et al. [32]	*Clin Physiol Funct Imaging* 2009; 29,:447	49	Dipy	TTE	No
Avakian et al. [33]	*IJC* 2001; 81: 21	110	Dipy	SPECT	No
Camuglia et al. [34]	*JACC* 2014; 63: 1808	10	IC adeno	Doppler wire	No
Vendrik et al. [35]	*JAHA* 2020; 9:e015133	13	IC adeno	FFR	No
Wiegerinck et al. [36]	*Circ CV Int* 2015; 8:e002443	27	IC adeno	Combo	No
Ahmad et al. [37]	*JACC CV Int* 2018; 11: 2019	28	IC adeno	FFR	No
Scarsini et al. [38]	*EuroIntervention* 2018; 13: 1512	66	IC adeno	FFR	No
Di Gioia et al. [39]	*AJC* 2016; 117: 1511	106	IC adeno	FFR	No
Scarsini et al. [38]	*J Cardiovasc Transl Res* 2019; 12: 539	82	IC/IV adeno	FFR	No
Stähli et al. [40]	*Cardiology* 2012; 123: 234	4	IV adeno	FFR	No
Stundl et al. [41]	*Clin Res Cardiol* 2019; 109	13	IV adeno	FFR	No
Lumley et al. [42]	*JACC* 2016; 68: 688	19	IV adeno	FFR	No
Burgstahler et al. [43]	*IJ CV Img* 2008; 24: 195	20	IV adeno	CMR	No
Hildick-Smith and Shapiro [44]	*JACC* 2000; 36: 1889	27	IV adeno	TTE	1 “tolerated poorly”
Mahmod et al. [45]	*JCMR* 2014; 16: 29	28	IV adeno	CMR	No
Samuels et al. [46]	*JACC* 1995; 25: 99	35	IV adeno	SPECT	2 hypotension, 2 AV block
Gutiérrez-Barrios et al. [47]	*Int J Cardiol* 2017; 236: 370	36	IV adeno	FFR	No
Stoller et al. [48]	*EuroIntervention* 2018; 14: 166	40	IV adeno	FFR	No
Takemoto et al. [49]	*JASE* 2014; 27: 200	41	IV adeno	TTE/FFR	No
Patsilinakos et al. [50]	*Angiology* 1999; 50: 309	50	IV adeno	TTE/SPECT	No
Stanojevic et al. [51]	*J Inv Card* 2016; 28: 357	72	IV adeno	FFR	No
Patsilinakos et al. [52]	*JNC* 2004; 11: 20	75	IV adeno	SPECT	9 AV block
Yamanaka et al. [53]	*JACC* CV Int 2018; 11: 2032	95	IV adeno	FFR/SPECT	1 AV block, 10% SBP < 40 mmHg
Ahn et al. [54]	*JACC* 2016; 67: 1412	117	IV adeno	CMR	No
Banovic et al. [55]	*Echo* 2014; 31: 428	127	IV adeno	TTE	No
Singh et al. [56]	*EHJ* 2017; 38: 1222	174	IV adeno	CMR	No
Nishi et al. [57]	*Coron Artery Dis* 2018; 29: 223	9	Mixed	FFR	No
Arashi et al. [58]	*Cardiovasc Interv Ther* 2019; 34: 269	13	Mixed	FFR	No
Hussain et al. [59]	*JNC* 2017; 24: 1200	95	Mixed	SPECT	No
Banovic et al. [60]	*Coron Artery Dis* 2020; 31: 166–73	4	NR	FFR	No
Cremer et al. [61]	*JNC* 2014; 21: 1001	50	Rega	PET	2 hypotension

AV = atrioventricular, adeno = adenosine, CMR = cardiac magnetic resonance, dipy = dipyridamole, FFR = fractional flow reserve, IC = intracoronary, IV = intravenous, *N* = number of subjects, NR = not reported, PET = positron emission tomography, rega = regadenoson, SBP = systolic blood pressure, SPECT = single-photon emission computed tomography, and TTE = transthoracic echocardiography.

**Table 2:** Literature review of hyperemic myocardial resistance with severe aortic stenosis.

Authors	Citation	Normal subjects			Aortic stenosis subjects						
		*N*	HMR	*p* value^*∗*^	*N*	Mean Δ*P* (mmHg)	AVA (cm^2^)	HMR	After TAVI	*p* value	Long term
*Doppler flow velocity with HMR in mmHg/(cm/sec) units*											
Vendrik et al. [35]	*JAHA* 2020; 9: e015133				13		0.83	2.54	2.18	<0.001†	1.95
Lumley et al. [42]	*JACC* 2016; 68: 688	30	2.29	0.14	19	57	0.74	2.82			
Wiegerinck et al. [36]	*Circ CV Int* 2015; 8: e002443	28	1.80	0.096	27	43	0.78	2.10	1.83	0.072	
Ahmad et al. [37]	*JACC CV Int* 2018; 11: 2019				30	38	0.68	2.42	2.14	0.03	

*Bolus thermodilution with HMR in mmHg* ^*∗*^ *sec units*											
Nishi et al. [57]	*Coron Artery Dis* 2018; 29: 223	30	16.2	0.14	9	54	0.70	20.4			
Gutiérrez-Barrios et al. [47]	*Int J Cardiol* 2017; 236: 370	10	17.8	0.01	36	53		32.7			
Stoller et al. [48]	*EuroIntervention* 2018; 14: 166				40	45 to 58‡	<1.0	26.6§	30.7	0.42	

∗ = compares normal versus aortic stenosis subjects. † = for this study, the *p* value refers to both Friedman test comparing baseline, post-TAVI, and long term as well as each pairwise comparison. ‡ = averages reported separately for subjects with (*N* = 26) and without (*N* = 14) coronary artery disease, respectively. § = only study to correct HMR using an explicitly measured zero-flow pressure. Δ*P* = pressure gradient, AVA = aortic valve area, HMR = hyperemic myocardial resistance, *N* = number of subjects, and TAVI = transcatheter aortic valve implantation.

**Table 3:** Review of ongoing trials of coronary revascularization in severe aortic stenosis.

Study acronym	Trial ID	Status	*N*	Description	Completion
FAVOR IV-QVAS	NCT03977129	Recruiting	792	Randomized comparison of QFR and angiography-guided revascularization	2022
NOTION-3	NCT03058627	Recruiting	452	Routine FFR-guided complete revascularization with PCI compared with conservative management in TAVI patients	2025
FAITAVI	NCT03360591	Recruiting	320	Comparison of angiography-guided versus physiology-guided PCI of patients with CAD undergoing TAVI	2021
TCW	NCT03424941	Recruiting	328	FFR-guided PCI and TAVI in severe AS and multivessel CAD vs. CABG and SAVR	2021
FORTUNA	NCT03665389	Not yet recruiting	25	Comparison of FFR derived from coronary computed tomography angiography before TAVR and FFR after TAVI	2022
None	NCT03442400	Recruiting	50	Comparison of pre- and post-TAVI iFR/FFR values and assessment of short-term outcomes	2019
TAVI-PCI	NCT04310046	Not yet recruiting	980	Comparison of FFR-guided PCI within 40 days before TAVI or within 40 days after TAVI	2023

**Table 4:** Literature review of coronary physiology before versus after treatment of severe aortic stenosis.

Authors	Citation	*N*	Baseline	Immediate	*p* value	Long term	*p* value	Time	Treatment	Method
Resting perfusion (cc/min/g) or Doppler velocity (cm/sec)										
Nemes et al. [28]	*Herz* 2002; 27: 780	21	62.2			40.1	<0.01	15 months	SAVR	Echo Doppler (diastolic)
Hildick-Smith and Shapiro [44]	*JACC* 2000; 36: 1889	27	43			41	NS	6 months	SAVR	Echo Doppler (diastolic)
Carpeggiani et al. [24]	*J CV Med* 2008; 9: 893	8	1.01			0.92	>0.05	12 months	SAVR	PET
Rajappan et al. [70]	*Circulation* 2003; 107: 3170	22	1.08			1.01	0.27	12 months	SAVR	PET
Camuglia et al. [34]	*JACC* 2014; 63: 1808	8	22	20	NS	18	NS	12 months	TAVI	Wire (Doppler)
Vendrik et al. [35]	*JAHA* 2020; pending	13	19.98	19.7	NS	21.44	0.397	6 months	TAVI	Wire (Doppler)
Ahmad et al. [37]	*JACC CV Int* 2018; 11: 2019	30	22.13	24.84	0.1				TAVI	Wire (Doppler)
Wiegerinck et al. [36]	*Circ CV Int* 2015; 8: e002443	27	24.4	25.5	0.401				TAVI	Wire (Doppler)
Instantaneous wave-free ratio (iFR)										
Vendrik et al. [35]	*JAHA* 2020; pending	13	0.82	0.83	NS	0.83	0.735	6 months	TAVI	
Ahmad et al. [37]	*JACC CV Int* 2018; 11: 2019	30	0.88	0.88	0.94				TAVI	
Scarsini et al. [38]	*EuroIntervention* 2018; 13: 1512	145	0.89	0.89	0.66				TAVI	
Hyperemic perfusion (cc/min/g) or Doppler velocity (cm/sec) or mean transit time (sec)										
Nemes et al. [28]	*Herz* 2002; 27: 780	21	117			91.5	<0.05	15 months	SAVR	Echo Doppler (diastolic)
Hildick-Smith and Shapiro [44]	*JACC* 2000; 36: 1889	27	71			108	<0.01	6 months	SAVR	Echo Doppler (diastolic)
Carpeggiani et al. [24]	*J CV Med* 2008; 9: 893	8	1.68			1.46	NS	12 months	SAVR	PET
Rajappan et al. [27]	*Circulation* 2003; 107: 3170	22	2.17			2.27	0.61	12 months	SAVR	PET
Camuglia et al. [34]	*JACC* 2014; 63: 1808	8	34	29	NS	39	NS	12 months	TAVI	Wire (Doppler)
Vendrik et al. [35]	*JAHA* 2020; pending	13	26.36	30.78	<0.001	40.2	<0.001	6 months	TAVI	Wire (Doppler)
Wiegerinck et al. [36]	*Circ CV Int* 2015; 8: e002443	27	44.5	51.1	0.027				TAVI	Wire (Doppler)
Ahmad et al. [37]	*JACC CV Int* 2018; 11: 2019	30	33.44	40.33	0.004				TAVI	Wire (Doppler)
Stoller et al. [48]	*EuroIntervention* 2018; 14: 166	40	0.44	0.48	0.53				TAVI	Wire (thermo)
Coronary flow reserve (CFR)										
Nemes et al. [32]	*Herz* 2002; 27: 780	21	1.96			2.37	<0.05	15 months	SAVR	Echo Doppler (diastolic)
Hildick-Smith and Shapiro [44]	*JACC* 2000; 36: 1889	27	1.76			2.61	<0.01	6 months	SAVR	Echo Doppler (diastolic)
Carpeggiani et al. [24]	*J CV Med* 2008; 9: 893	8	1.68			1.58	NS	12 months	SAVR	PET
Rajappan et al. [70]	*Circulation* 2003; 107: 3170	22	2.02			2.28	0.17	12 months	SAVR	PET
Camuglia et al. [34]	*JACC* 2014; 63: 1808	8	1.53	1.58	0.41	2.18	<0.01	12 months	TAVI	Wire (Doppler)
Vendrik et al. [35]	*JAHA* 2020; pending	13	1.28	1.65	<0.001	1.94	<0.001	6 months	TAVI	Wire (Doppler)
Wiegerinck et al. [36]	*Circ CV Int* 2015; 8: e002443	27	1.9	2.1	0.113				TAVI	Wire (Doppler)
Stoller et al. [48]	*EuroIntervention* 2018; 14: 166	40	1.9	2	0.72				TAVI	Wire (thermo)
Fractional flow reserve (FFR)										
Stundl et al. [41]	*Clin Res Cardiol* 2019; Epub	13	0.77			0.76	0.11	2 months	TAVI	
Vendrik et al. [35]	*JAHA* 2020; pending	13	0.85	0.79	<0.001	0.71	<0.001	6 months	TAVI	
Ahmad et al. [37]	*JACC CV Int* 2018; 11: 2019	30	0.87	0.85	0.0008				TAVI	
Stoller et al. [48]	*EuroIntervention* 2018; 14: 166	40	0.9	0.93	0.0021				TAVI	
Pesarini et al. [71]	*Circ CV Int* 2016; 9: e004088	133	0.89	0.89	0.73				TAVI	

NS = not significant (actual *p* value not reported), PET = positron emission tomography, SAVR = surgical aortic valve replacement, TAVI = transcatheter aortic valve implantation, and thermo = bolus thermodilution (based on the table from 2020 editorial [68]).
